# Cardiovascular equity and health center funding: Associations of unmet hypertension and diabetes need by race/ethnicity and federal grants at federally qualified health centers, 2014–2019

**DOI:** 10.1371/journal.pone.0310523

**Published:** 2024-09-18

**Authors:** Sanjay Kishore, Sandeep P. Kishore, Cheryl Clark, Benjamin D. Sommers

**Affiliations:** 1 Department of Medicine, University of Virginia, Charlottesville, Virginia, United States of America; 2 Department of Medicine, University of California San Francisco, San Francisco, California, United States of America; 3 Department of Medicine, Brigham and Women’s Hospital, Boston, Massachusetts, United States of America; 4 Department of Health Policy and Management, Harvard T.H. Chan School of Public Health, Boston, Massachusetts, United States of America; Dayeh University, TAIWAN

## Abstract

**Importance:**

Racial and ethnic disparities in chronic disease are a major public health priority.

**Objective:**

To determine if the amount of federal grant funding to federally-qualified health centers (FQHCs) was associated with baseline overall prevalence of uncontrolled hypertension and uncontrolled diabetes, as well as prevalence by racial and ethnic subgroup.

**Design:**

Cross-sectional multivariate regression analysis of Uniform Data System 2014–2019, which includes clinic-level data from each FQHC regarding demographics, chronic disease control by race and ethnicity, and grant funding.

**Exposures:**

Our main exposure were the average values of the prevalence of uncontrolled hypertension and uncontrolled diabetes among the overall population and by racial and ethnic group from 2014–2016.

**Main outcomes:**

Average federal grant funding per patient from 2017–2019, as measured by annual health center funding from the Bureau of Primary Health Care (BPHC) and overall federal grant funding.

**Results:**

We analyzed 1,205 FQHCs from 2014–2019; the average BPHC grant per patient across all FQHCs in 2019 was $168 while the average total federal grant was $184 per patient. Increasing shares of total patients with uncontrolled hypertension or uncontrolled diabetes were not associated with increased total federal grant funding in either unadjusted or adjusted analysis. Increased shares of patients who are American Indian or Alaskan Native (AI-AN) with uncontrolled hypertension and diabetes were associated with increasing total federal grant funding in both unadjusted and adjusted analysis (adjusted beta hypertension $168.3, p <0.001; adjusted beta diabetes 59.44, p = 0.02). However, cardiovascular clinical need among other racial and ethnic groups was not significantly associated with grant funding.

**Conclusions:**

FQHCs with higher overall rates of uncontrolled hypertension or diabetes do not receive more federal funds, and there is no significant association between federal funding levels and rates of uncontrolled blood pressure or diabetes within most racial and ethnic groups, with the exception of AI-AN populations. To narrow inequities in cardiovascular disease, HRSA should consider more explicitly targeting federal grants to clinics with higher levels of clinical need.

## Introduction

Cardiovascular disease remains the leading cause of death for Americans, with large inequities in who faces morbidity and mortality along lines of race and class. Experts have identified structural racism as a driver for these differences in outcomes [1]. A critical target for reform is improving both access and quality of primary care for those who face the highest cardiovascular risk.

Federally-qualified health centers (FQHCs) are the cornerstone of the primary care safety-net in the United States, providing access to care for over 30 million predominantly low-income Americans in the past year. A core aim of FQHCs is to expand high-quality preventive care to all adults, including the diagnosis and treatment of key cardiovascular risk factors such as hypertension and diabetes.

As in other health systems, chronic disease management remains a challenge in federal health centers. In 2019, rates of blood pressure control (defined as BP < 140/90 for patients with hypertension) were 60.1% in FQHCs. This was far short of the 80% target promoted by initiatives such as the Million Hearts Campaign sponsored by the Department of Health and Human Services [2].

These population-level measures mask dramatic differences within racial and ethnic groups, however. Across the United States, Non-Hispanic Black patients have among the highest burden of cardiovascular disease, and these patterns hold true within the FQHC patient population as well [3]. These differences in clinical outcomes may be exacerbated by structural determinants of health–including poverty, insurance status, and access to providers–but do not explain all variation [4].

For years, federal agencies and public health stakeholders have highlighted the urgency of addressing these inequities. The Centers for Disease Control and Prevention (CDC) has declared racism as a threat to public health and called for the elimination of health disparities between racial and ethnic groups to be a guiding principle of its policy and programs moving forward [5]. Similarly, the former Surgeon General issued a Call to Action to Control Hypertension prioritizing the elimination of disparities between racial and ethnic groups in the ability to receive adequate blood pressure treatment [6].

A potential lever to help remedy disparities within the FQHC program is grant funding. FQHCs rely on federal grants to provide quality care and offset the costs of caring for patients who are in poverty and are often uninsured or underinsured. Annual grants are allocated primarily through the Health Resources and Services Administration (HRSA) Bureau of Primary Health Care (BPHC), as authorized through Section 330 of the US Public Health Service Act, and are generally distributed in a competitive application process. Beyond HRSA, other federal agencies are also able to offer grants to help FQHCs respond to specific public health priorities. In 2022, FQHCs received over $9 billion in federal grants [7].

Given the role of health centers as primary care providers for millions of low-income Americans, our objective was to assess whether there is any association between measures of clinical need and HRSA grants. More specifically, do FQHCs serving patients with higher levels of need (especially among racial and ethnic communities that have traditionally faced higher cardiovascular burden of disease) receive greater funding amounts per patient in future years than other centers?

In this analysis, we explore this question using publicly-available data from the Health Resources and Services Administration (HRSA). We define clinical need in terms of two measures: uncontrolled diabetes and uncontrolled hypertension by racial and ethnic group.

## Methods

We used data from the 2014–2019 Uniform Data Services (UDS) accessed December 2023 [8]. The UDS describes all FQHC grantees and includes clinic-level demographic, clinical, and financial characteristics that are reported to HRSA annually and are publicly-available. We include UDS data from all U.S. states and excluded those in U.S. territories for the purposes of this analysis.

We focused on these years as the quality of data reported in 2020 and years thereafter was less complete and may have been affected by the COVID pandemic. Additionally, federal financing to FQHCs also changed in response to the pandemic; for instance, the Biden Administration allocated over $6 billion to health centers through the American Rescue Plan in 2021 to help stand up COVID testing and vaccination delivery infrastructure [9].

Outcome variables were averages of two separate measures of federal grants from 2017–2019: Bureau of Primary Health Care grants (BPHC), and total federal grants as reported in the UDS (including non-BPHC grants and other sources), both expressed in terms of dollars per patient.

Independent variables were all FQHC-level measures based on average values of demographic and clinical variables from 2014–2016. Our key exposure variables were measures of clinical need, defined as the percent of each clinic’s population that had uncontrolled hypertension or uncontrolled diabetes overall and by racial and ethnic subgroup. For instance, to derive a measure of blood pressure control among Non-Hispanic Black patients at a health center, we took the absolute number of Non-Hispanic Black patients reported to have uncontrolled hypertension, divided this by the total number of health center patients.

We constructed our measure this way to capture a definition of clinical need that reflected both the prevalence of hypertension or diabetes in the FQHC patient population as well as the share of that cohort without adequate clinical control. An alternative measure, such as a measure of control among those patients with hypertension or diabetes, would not reflect the baseline prevalence of these conditions, which we believe is relevant to how need is assessed. We include a sensitivity analysis examining separate terms for prevalence and control of both hypertension and diabetes in the Supplement.

We then standardized this measure, such that clinics with worse hypertension or diabetes control would be identified as those with an increase of one standard deviation of the percent of all patients within each racial or ethnic group with uncontrolled blood pressure or hemoglobin A1c.

We used clinical data from 2014–2016 to predict funding levels over 2017–2019 to avoid potential reverse causality—namely, whether higher levels of grant funding in the past reduced rates of uncontrolled chronic disease in the future. Our approach attempts to mirror how funding decisions could reasonably be made by looking at previous clinical and demographic measures to make funding decisions going forward.

We included information about all racial/ethnic groups that had less than 10% missing data in each year from FQHCs: Hispanic, Non-Hispanic Black, Non-Hispanic White, Asian, and American Indian/Alaskan Native (AI/AN); we did not include Native Hawaiian and Pacific Islanders (NHPI) due to missingness (more detail on missing data is included in the supplement).

We also adjusted for the following FQHC-level covariates: the total number of patients at each clinic (expressed in thousands) and demographics such as the percentage of patients with incomes under the 100% federal poverty level, percent uninsured, and percent of patients by racial and ethnic group.

We used linear regression in two separate models for each outcome variable on our set of predictors, and reported both unadjusted and adjusted regression analysis. In the unadjusted analysis, each outcome variable was regressed on each domain of predictors (e.g. demographic terms only including racial/ethnic composition, uncontrolled hypertension terms only including percent overall uncontrolled blood pressure and by racial/ethnic group, uncontrolled diabetes terms only including percent overall uncontrolled hemoglobin A1c and by racial/ethnic group) whereas in the adjusted analysis each outcome was regressed on all predictors.

We included data from the 1,205 FQHC grantees that reported data for all six years within our time horizon, representing 86% of the unique health centers in the UDS databases from 2014–2019. After excluding data from racial/ethnic groups that had over 10% missing data, the percentage of missing values across the predictor variables varied from 0 to 8% per year. There were no missing values for BPHC grants 2017, 2018, and 2019, and there were no missing values for total federal grants in 2018 and 2019.

Statistical significance was defined based on a two-sided alpha of 0.05. All analysis was completed in R (R Core Team, 2022). This study was exempt from IRB review given the use of publicly-available data. Sensitivity analyses including separate measures of chronic disease prevalence and control, as well as of FQHCs stratified by rates of uninsurance, are included in an online supplement (S1 Appendix).

## Results

**Fig 1:**
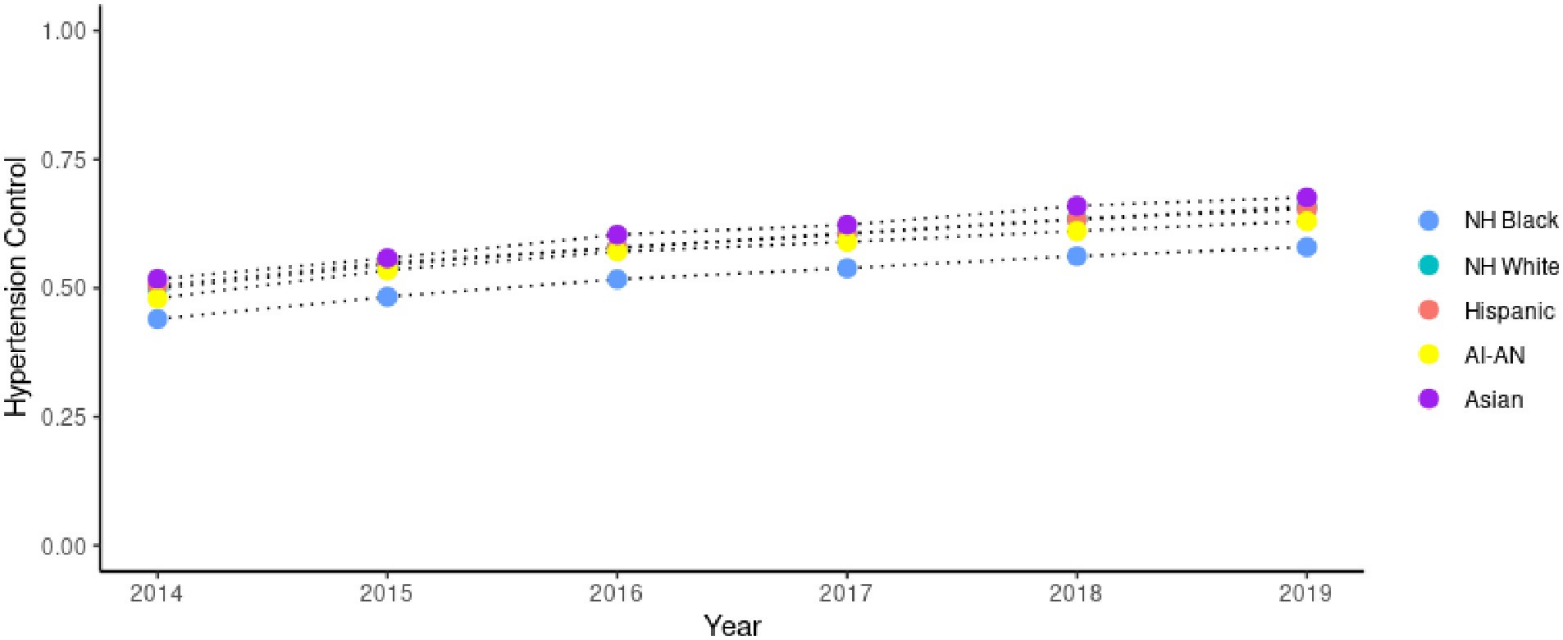
Rates of hypertension control among FQHCs 2014–2019 by race/ethnicity. Source: Uniform Data Services 2014–2019.

**Fig 2:**
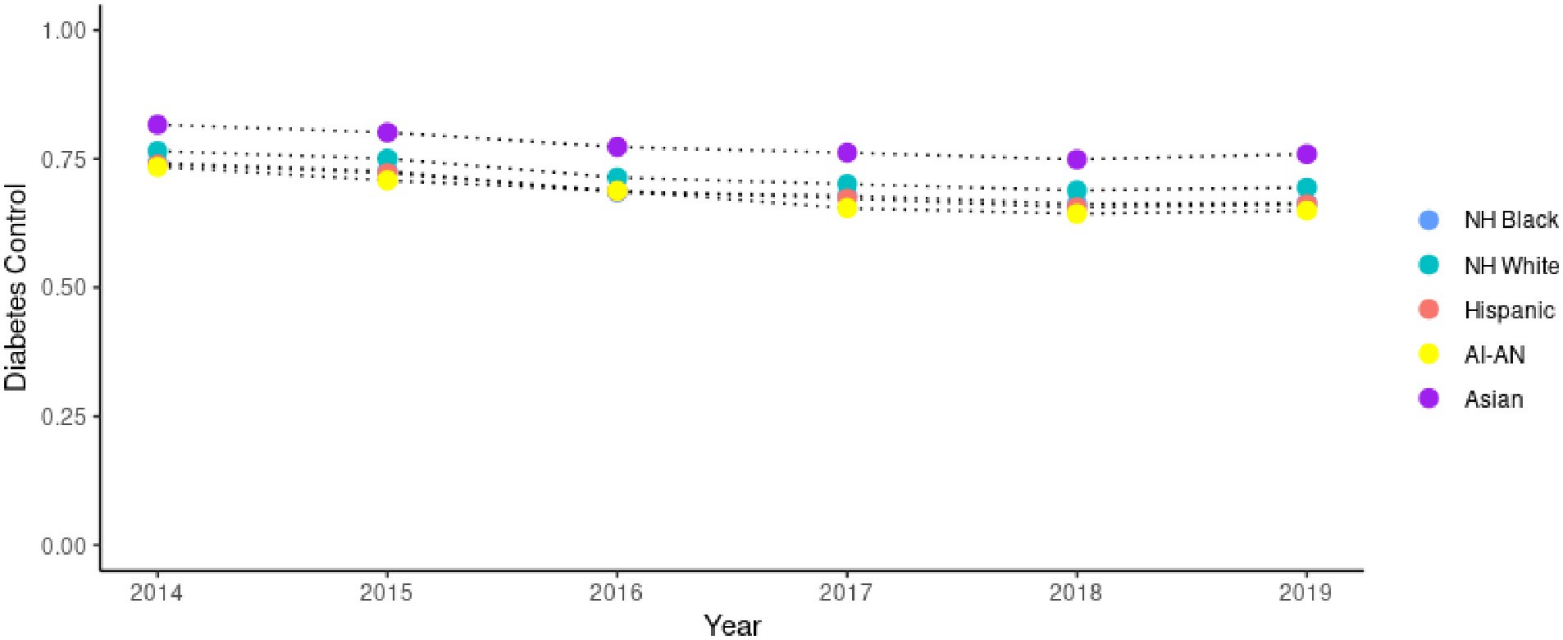
Rates of diabetes control among FQHCs 2014–2019 by race/ethnicity. Source: Uniform Data Services 2014–2019.

Our analysis includes data from 1,205 unique FQHCs with demographic and clinical characteristics by year from 2014–2019 displayed in Table 1. The average percent of all patients within the FQHC total patient population with uncontrolled hypertension ranged from 7.1% in 2014 to 5.9% in 2019, and for uncontrolled diabetes ranged from 1.7% in 2014 to 2.7% in 2019. The average BPHC grant per patient across all FQHCs in 2019 was $168, while the average total federal grants were $184 per patient. Figs 1 and 2 display differences in hypertension control and diabetes control by racial/ethnic group in FQHCs throughout recent years, respectively.

**Table 1:** Descriptive characteristics of FQHCs, 2014–2019.

Year	2014	2015	2016	2017	2018	2019
Total Patients	22,159,608	23,196,517	24,647,337	25,809,864	26,848,951	27,963,898
Uninsured	27.88	24.39	23.42	22.91	22.58	22.77
Poverty	52.45	51.76	50.47	49.52	48.60	48.26
% NH White	36.55	36.30	36.38	36.03	35.83	35.60
% NH Black	19.62	19.32	18.84	18.73	18.33	17.79
% Hispanic	33.17	33.35	33.48	33.87	34.10	34.38
% Asian	3.24	3.34	3.42	3.54	3.56	3.56
% AI-AN	0.98	0.96	0.97	0.94	0.91	0.89
% Overall HTN Uncontrolled	7.09	6.37	6.67	6.48	6.09	5.90
% NH Black HTN Uncontrolled	2.03	1.86	1.91	1.89	1.78	1.73
% NH White HTN Uncontrolled	2.88	2.53	2.65	2.55	2.36	2.27
% Hispanic HTN Uncontrolled	1.60	1.47	1.56	1.51	1.43	1.38
% Asian HTN Uncontrolled	0.21	0.21	0.21	0.21	0.19	0.19
% AI-AN HTN Uncontrolled	0.06	0.05	0.06	0.06	0.05	0.05
% Overall DM Uncontrolled	1.70	1.78	2.35	2.55	2.71	2.66
% NH Black DM Uncontrolled	0.38	0.40	0.55	0.59	0.61	0.58
% NH White DM Uncontrolled	0.58	0.57	0.75	0.79	0.82	0.81
% Hispanic DM Uncontrolled	0.59	0.65	0.85	0.96	1.04	1.02
% Asian DM Uncontrolled	0.04	0.05	0.06	0.07	0.07	0.07
% AI-AN DM Uncontrolled	0.02	0.02	0.03	0.03	0.03	0.03
BPHC Grants (Per Patient)	$139.35	$152.98	$169.68	$173.51	$170.41	$168.85
Total Federal Grants (Per Patient)	$160.06	$169.54	$183.25	$188.41	$185.68	$184.40

Source: Uniform Data Services, 2014–2019

Notes: Values for each racial/ethnic group with uncontrolled hypertension are reported as percent of the total FQHC patient population. Each value (except for number of total patients) reflect the weighted mean among the 1,205 FQHCs in the sample.

With respect to our primary independent variables, we did not observe an association between overall levels of both uncontrolled hypertension/diabetes and federal grants in either unadjusted analysis or adjusted analysis. Results from our regression analysis are presented in Table 2.

**Table 2:** Regression analysis of per capita federal grants by FQHC characteristics.

		Unadjusted BPHC		Adjusted BPHC		Unadjusted Total Federal		Adjusted Total Federal	
	Mean	Beta	p-value	Beta	p-value	Beta	p-value	Beta	p-value
**Demographics**									
**# Patients (Thousands)**	19.365	-3.171	<0.001	-3.041	<0.001	-3.364	<0.001	-3.188	<0.001
% Poverty	48.267	-0.517	0.347	-0.466	0.396	-0.459	0.529	-0.475	0.511
**% Uninsured**	27.808	3.997	<0.001	3.932	<0.001	4.118	<0.001	4.189	<0.001
% Hispanic	25.154	-0.738	0.154	-0.77	0.434	-0.566	0.41	-0.528	0.684
% Non-Hispanic Black	19.573	0.167	0.746	0.86	0.426	0.489	0.475	1.225	0.39
% Asian	3.101	-0.523	0.648	-0.974	0.643	-0.348	0.819	-1.092	0.694
% American-Indian/Alaskan Native	2.285	10.322	<0.001	2.159	0.329	18.252	<0.001	-0.2	0.945
% Other Racial/Ethnic Group	6.668	2.906	0.043	2.77	0.055	3.34	0.08	3.312	0.082
**Hypertension**									
% All HTN Uncontrolled	0	10.722	0.456	0.609	0.975	0.754	0.968	-0.4	0.987
% Hispanic HTN Uncontrolled	0	-2.27	0.849	10.643	0.552	0.279	0.986	9.769	0.679
% NH Black HTN Uncontrolled	0	5.667	0.69	-14.007	0.593	14.22	0.441	-15.194	0.66
% Asian HTN Uncontrolled	0	-4.779	0.672	5.654	0.736	-2.127	0.885	8.886	0.688
**% AI-AN HTN Uncontrolled**	0	136.122	<0.001	143.313	<0.001	225.261	<0.001	168.314	<0.001
**Diabetes**									
% All DM Uncontrolled	0	37.673	0.051	31.759	0.19	33.119	0.181	32.943	0.302
% Hispanic DM Uncontrolled	0	-26.417	0.09	-15.952	0.52	-22.753	0.255	-19.491	0.55
%NH Black DM Uncontrolled	0	-5.349	0.744	-16.71	0.513	3.257	0.877	-16.891	0.616
% Asian DM Uncontrolled	0	-1.329	0.911	-0.395	0.98	1.857	0.904	0.834	0.968
**% AI-AN DM Uncontrolled**	0	87.405	<0.001	-48.883	0.013	196.525	<0.001	59.44	0.021

Source: Uniform Data Services, 2014–2019

Notes: Hypertension and diabetes variables are standardized. Betas for these variables should be interpreted as the unadjusted or adjusted effect of an increase in a standard deviation of the percent of uncontrolled blood pressure or diabetes within a racial/ethnic subgroup. Those variables marked in red have significant associations (p < 0.05) in both unadjusted and adjusted analysis for either Bureau of Primary Health Care Grants or Total Federal Grants. Unadjusted analyses examine the association of federal grants with all terms within each domain of predictors (demographics, hypertension, diabetes); adjusted analyses examine the association of federal grants with all predictors.

The only racial/ethnic subgroup with an association between increased shares of uncontrolled hypertension and diabetes and funding was American Indian and Alaska Natives (AI-AN). Clinics with higher shares of AI-AN patients with uncontrolled blood pressure were associated with $143.31 more per patient in BPHC grants (p <0.001) and $168.31 in total federal grants per patient (p < 0.001) in adjusted analysis. Clinics with increasing shares of AI-AN patients with uncontrolled diabetes were associated with $48.88 less per patient in BPHC grants (p = .01) but $59.33 more in overall federal grants (p = 0.02). We note that the beta coefficient changed from positive to negative when adjusted for all covariates (unadjusted beta $87.40, p < 0.001; adjusted beta -$48.88, p = 0.01). On further analysis, this was explained by inclusion of the percent population AI-AN; when this term was removed, the coefficient was positive.

**Fig 3:**
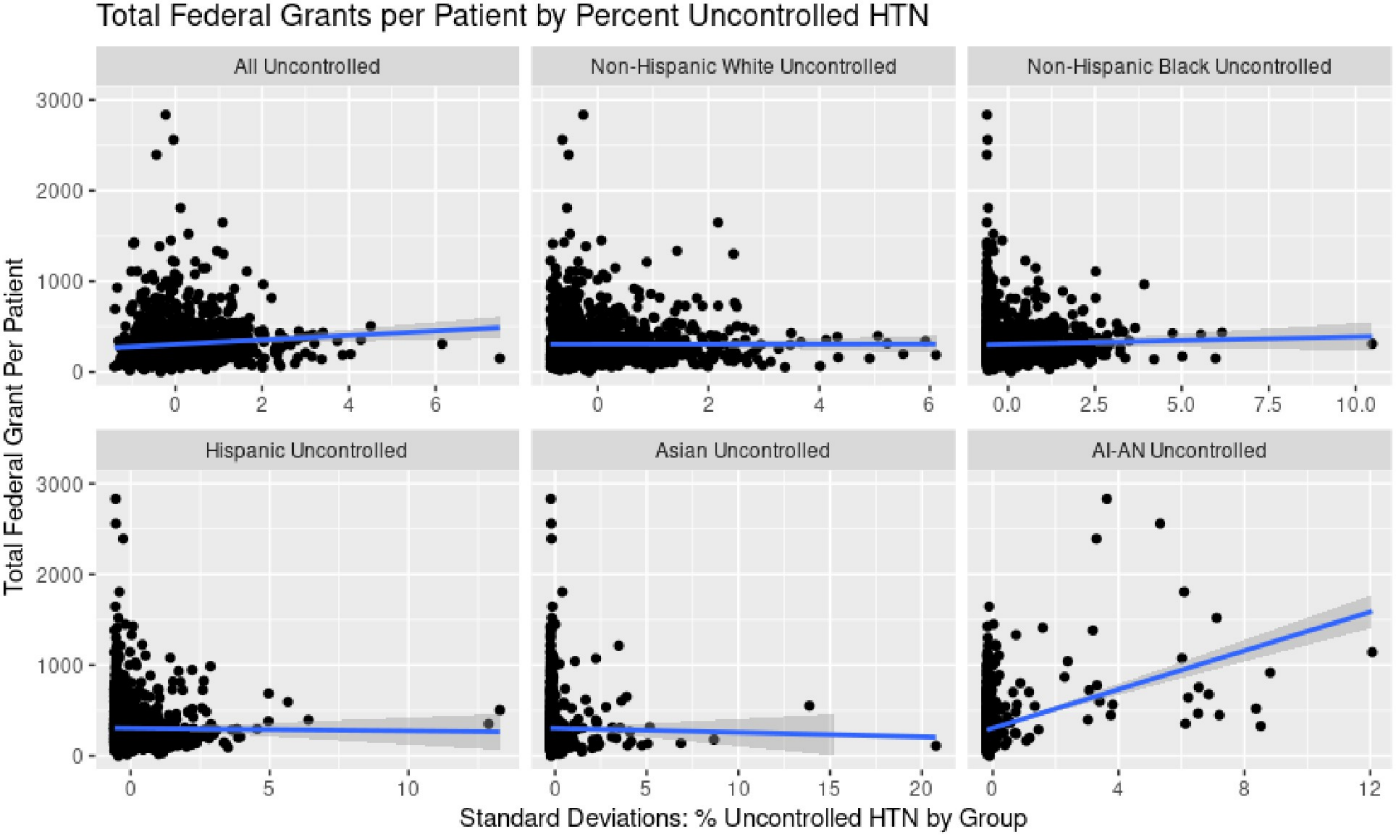
Association of average percent patients by racial/ethnic subgroup with uncontrolled hypertension (2014–2016) and federal funding (2017–2019).

**Fig 4:**
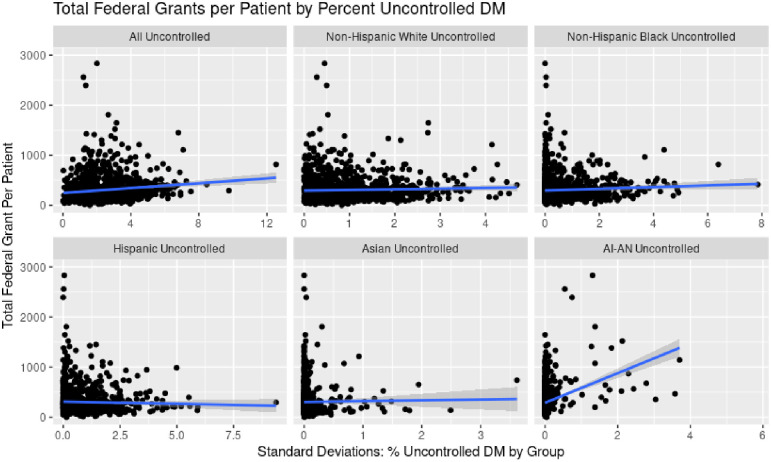
Association of average percent patients by racial/ethnic subgroup with uncontrolled diabetes (2014–2016) and federal funding (2017–2019).

Importantly, clinics with higher shares of patients with uncontrolled hypertension or diabetes among Black, Hispanic, or Asian populations were not associated with BPHC nor total federal grants in either unadjusted or adjusted analysis. Figs 3 and 4 show a scatterplot of increasing shares of uncontrolled hypertension and diabetes respectively by racial/ethnic group and total federal grants.

Notably, the number of patients and percent uninsured were associated with at least one source of funding in both unadjusted and adjusted analysis. For every increase of 1,000 patients in the average population served by each clinic, BPHC grants decreased by $3.04 per patient (p <0.001), and federal grants by $3.18 (p < 0.001) in adjusted analysis. For every percentage-point increase in the uninsured population served by each clinic, BPHC grants increased by $3.93 (p<0.001) and total federal grants by $4.19 (p<0.001) in adjusted analysis.

## Discussion

In this analysis of all federally-qualified health centers from 2014–2019, we set out to understand if there was any relationship between levels of cardiovascular clinical need (defined as the share of all FQHC patients with uncontrolled hypertension or diabetes) and federal funding in subsequent years. We did not observe an association between levels of uncontrolled hypertension or diabetes and federal funding among both overall FQHC patient population and within most racial/ethnic subgroups, with the exception of American-Indian/Alaskan Native populations.

Our study addresses a key gap in the literature. While FQHCs are a pillar of the primary care network for the most marginalized Americans, little research has examined whether those clinics with the highest level of clinical need receive more federal investment than others.

FQHCs are funded through a competitive grant application process in which clinical outcomes are generally not an explicit criterion [10]. Our analysis does not show a consistent relationship between key measures of primary care need and financing.

Moreover, despite an increasing policy focus on addressing racial disparities in chronic disease, we do not find consistent evidence for a tie between FQHC funding and measures of clinical need among several historically marginalized groups such as Black and Hispanic populations. Clinical measures of need for AI-AN individuals did track with funding levels in most models, which may in part reflect funding levels for the 36 FQHCs with large AI-AN populations (> 20% AI-AN population share), particularly common in Alaska. But for other groups, including Black and Hispanic patients, the lack of an association may indicate a missed opportunity to direct funding to populations exposed to greater health risks based on a variety of inequities associated with structural racism, including housing, income, nutrition, and education.

Though many factors contribute to varying rates of control of chronic disease among racial/ethnic groups, clinical inertia is a key barrier. Appropriate intensification of blood pressure therapy occurred less than 11% of the time when indicated as per American Heart Association guidelines in older adults [11]. Roughly 40% of patients with hypertension are only on monotherapy, even though guidelines suggest the majority of patients should be on combination therapy [12]. Scholars have named lack of treatment intensification as a major missed opportunity for narrowing health inequities [13].

Other systems have demonstrated that gaps in hypertension and diabetes can be addressed through population health strategies ranging from standardized protocols, patient registries, and team-based care utilizing pharmacists, community health workers, and other professionals in the primary care setting [14, 15].

However, these interventions require resources. Federal funds subsidize staffing, systems, and enabling services (including medication assistance, transportation, and care coordination) that improve outcomes at FQHCs [16]. Moreover, FQHCs in states that failed to expand Medicaid rely more heavily on federal grants to sustain their operations as they could not depend on as much insurance reimbursement [17].

In this context, our findings raise the question of whether federal grant funding for FQHCs should be tied to clinical outcomes, particularly for historically marginalized populations. As the field of primary care evolves to recognize the merits of value-based care and the role of the social determinants of health, we should at the very least consider a financing structure for FQHCs that more explicitly allocates resources with an eye towards need.

Second, if advancing equity and reducing disparities in chronic disease management are a major priority, policymakers should consider prioritizing federal funding to those groups that are both historically marginalized and have the highest burden of disease. This framing is consistent with how the Biden-Harris Administration viewed the responsibility of government in an Executive Order on Advancing Racial Equity and Support for Underserved Communities in 2021, as well as the Department of Health & Human Services’ Equity Action Plan [18, 19].

We note that our analysis comes as HRSA has been working to incorporate broader measures of need in their financing of new sites, known as the “Unmet Need Score” [20]. This measure includes socioeconomic status indices and variables for prevalence of disease (e.g. diabetes, asthma, smoking), but does not include measures for control of disease. At this time, it is not clear if this will be used for allocating existing grant funds to each FQHC.

Our analysis is limited by its observational nature and as such we cannot infer causality. While prior health center performance may be associated with future performance, we aimed to understand whether variation in grant funding was responsive to clinical need. Though autocorrelation among both exposures and outcome variables is possible, we attempted to mitigate this through a three-year period for exposures and then a subsequent three-year period for outcomes.

Moreover, our findings should be contextualized in the setting of blood pressure control being defined liberally (BP < 140/90 for patients with hypertension), compared to recent clinical standards defining control as BP < 130/80. This means overall rates of control could be even lower than as reported here, and possibly disparities worsened. Future research including qualitative methods may shed additional insights into how FQHC grant dollars can be used to address these clinical needs, and match grant funding levels commensurate with unmet needs to eliminate disparities and improve health outcomes over time.

In conclusion, we do not find that grant funding to FQHCs is associated with measures of uncontrolled hypertension or diabetes for overall patient populations or most racial/ethnic subgroups. While higher rates of clinical need among AI-AN patients is associated with more financial support, we do not find that federal grants are associated with the clinical needs of Black, Hispanic, and Asian patients, suggesting a potential missed opportunity to leverage federal funding to address historical health disparities in these populations.

## Supporting information

S1 Appendix (DOCX)
