# Supplementary material for: Cardiovascular equity and health center funding: Associations of unmet hypertension and diabetes need by race/ethnicity and federal grants at federally qualified health centers, 2014–2019
Source: PLoS One. 2024 Sep 18;19(9):e0310523. doi: 10.1371/journal.pone.0310523 (PMC11410232; doi:10.1371/journal.pone.0310523)
Supplement: S1 Appendix — (DOCX) [file pone.0310523.s001.docx]

**S1 Appendix**

**Number of Years Each FQHC Reported Data to UDS 2014-2019**

There were 1,398 unique FQHC organizations that reported data between 2014 and 2019. Of these, 1,205 reported data for all six years. We restricted our sample to these clinics.

| Number of Years Data Reported in UDS 2014-2019 | FQHCs |
| --- | --- |
| 1 | 36 |
| 2 | 11 |
| 3 | 24 |
| 4 | 14 |
| 5 | 108 |
| 6 | 1,205 |

**Proportion of Clinics with Missing Data for Clinical Measures**

***Note more than 10% of FQHCs missing data for Native-Hawaiian/Pacific-Islander in 2015-2019 and that prevalence of missing data increases across most categories 2020-2022**

| **Year** | 2014 | 2015 | 2016 | 2017 | 2018 | 2019 | 2020 | 2021 | 2022 |
| --- | --- | --- | --- | --- | --- | --- | --- | --- | --- |
| **# Clinic** | 1,249 | 1,345 | 1,337 | 1,342 | 1,331 | 1,352 | 1343 | 1341 | 1338 |
| **% White HTN Uncontrol** | 0 | 0.01 | 0.01 | 0.00 | 0.01 | 0.01 | 0.05 | 0.04 | 0.04 |
| **% Black HTN Uncontrol** | 0 | 0.03 | 0.01 | 0.01 | 0.01 | 0.01 | 0.24 | 0.22 | 0.22 |
| **% Hispanic HTN Uncontrol** | 0 | 0.02 | 0.01 | 0.00 | 0.00 | 0.00 | 0.17 | 0.15 | 0.15 |
| **% NHPI HTN Uncontrol** | 0 | 0.15 | 0.13 | 0.11 | 0.13 | 0.12 | 0.73 | 0.75 | 0.73 |
| **%Asian HTN Uncontrol** | 0 | 0.06 | 0.03 | 0.03 | 0.03 | 0.03 | 0.54 | 0.5 | 0.5 |
| **% AIAN HTN Uncontrol** | 0 | 0.08 | 0.05 | 0.04 | 0.04 | 0.04 | 0.71 | 0.69 | 0.68 |
| **%White DM Uncontrol** | 0 | 0.02 | 0.01 | 0.01 | 0.01 | 0.01 | 0.13 | 0.14 | 0.13 |
| **%NH Black DM Uncontrol** | 0 | 0.03 | 0.02 | 0.02 | 0.01 | 0.01 | 0.32 | 0.32 | 0.32 |
| **% Hispanic DM Uncontrol** | 0 | 0.02 | 0.01 | 0.00 | 0.00 | 0.00 | 0.26 | 0.26 | 0.24 |
| **% NHPI DM Uncontrol** | 0 | 0.13 | 0.14 | 0.11 | 0.15 | 0.13 | 0.53 | 0.52 | 0.53 |
| **% Asian DM Uncontrol** | 0 | 0.06 | 0.05 | 0.04 | 0.04 | 0.04 | 0.55 | 0.56 | 0.57 |
| **% AIAN DM Uncontrol** | 0 | 0.08 | 0.06 | 0.04 | 0.05 | 0.06 | 0.63 | 0.63 | 0.62 |
| **BPHC Grants** | 0 | 0.01 | 0.00 | 0.00 | 0.00 | 0.00 | 0 | 0 | 0 |
| **Total Federal Grants** | 0 | 0.14 | 0.15 | 0.14 | 0.00 | 0.00 | 0 | 0 | 0 |

**Clinics with High American-Indian and Alaskan-Native Population Share**

*Sorted by % AI-AN population share, including measure of chronic disease (standard deviations of percent of AI-AN patients with uncontrolled hypertension or uncontrolled diabetes) and federal grant funding (per patient).*

|  | **Clinic** | **City** | **State** | **% AI-AN** | **% AI-AN HTN Uncontrol** | **%AI-AN DM Uncontrol** | **BPHC Grant** | **Total Federal Grant** |
| --- | --- | --- | --- | --- | --- | --- | --- | --- |
| **1** | TUBA CITY REGIONAL HEALTH CARE CORPORATION | Tuba City | AZ | 99.18 | 5.56 | 5.39 | $296.42 | $323.79 |
| **2** | YUKON KUSKOKWIM HEALTH CORPORATION | Bethel | AK | 96.78 | 2.77 | 1.19 | $504.47 | $501.45 |
| **3** | COUNCIL OF ATHABASCAN TRIBAL GOVERNMENT | Fort Yukon | AK | 92.73 | 12.57 | 12.85 | $1,155.47 | $1,402.23 |
| **4** | PUEBLO OF JEMEZ | Jemez Pueblo | NM | 90.36 | 8.74 | 19.06 | $425.78 | $3,894.92 |
| **5** | NORTON SOUND HEALTH CORPORATION | Nome | AK | 89.84 | 3.81 | 0.52 | $268.77 | $452.50 |
| **6** | MANILLAQ ASSOCIATION | Kotzebue | AK | 89.54 | 8.18 | 3.23 | $328.26 | $328.26 |
| **7** | BRISTOL BAY AREA HEALTH CORPORATION | Dillingham | AK | 77.69 | 7.97 | 5.61 | $481.96 | $481.96 |
| **8** | TANANA CHIEFS CONFERENCE | FAIRBANKS | AK | 76.59 | 6.38 | 2.18 | $706.08 | $706.08 |
| **9** | TANANA CHIEFS CONFERENCE | Fairbanks | AK | 76.59 | 6.38 | 2.18 | $706.08 | $706.08 |
| **10** | BETHEL FAMILY CLINIC | Bethel | AK | 74.57 | 0.07 | 0.15 | $565.19 | $565.88 |
| **11** | COLVILLE CONFEDERATED TRIBES | Inchelium | WA | 72.41 | 6.27 | 8.10 | $456.25 | $1,130.09 |
| **12** | UTAH NAVAJO HEALTH SYSTEM, INC. | Montezuma Creek | UT | 70.49 | 6.89 | 10.10 | $283.04 | $368.73 |
| **13** | ALEUTIAN PRIBILOF ISLAND ASSOCIATIONS | ANCHORAGE | AK | 66.10 | 8.08 | 5.31 | $12,479.83 | $17,124.44 |
| **14** | ALEUTIAN PRIBILOF ISLAND ASSOCIATIONS | Anchorage | AK | 66.10 | 8.08 | 5.31 | $12,479.83 | $17,124.44 |
| **15** | SEATTLE INDIAN HEALTH BOARD INC | Seattle | WA | 60.36 | 2.15 | 6.51 | $402.40 | $1,135.51 |
| **16** | NATIVE AMERICAN COMMUNITY CLINIC | Minneapolis | MN | 59.62 | 3.02 | 4.26 | $397.80 | $405.87 |
| **17** | KODIAK AREA NATIVE ASSOCIATION | Kodiak | AK | 59.30 | 3.26 | 1.49 | $582.56 | $747.21 |
| **18** | N. A. T. I. V. E. PROJECT, THE | Spokane | WA | 57.93 | 6.97 | 2.68 | $371.43 | $897.44 |
| **19** | SOUTHCENTRAL FOUNDATION | Anchorage | AK | 56.25 | 4.54 | 1.64 | $2,711.00 | $2,334.01 |
| **20** | BIGHORN VALLEY HEALTH CENTER | Hardin | MT | 54.73 | 4.96 | 8.17 | $736.36 | $755.24 |
| **21** | NATIVE AMERICAN REHABILITATION ASSOCIATION OF THE NORTHWEST | Portland | OR | 54.41 | 7.85 | 5.65 | $371.78 | $1,728.88 |
| **22** | YAKUTAT TLINGIT TRIBE | Yakutat | AK | 54.17 | 3.35 | 1.68 | $2,156.47 | $2,455.73 |
| **23** | BAY MILLS INDIAN COMMUNITY | Brimley | MI | 52.73 | 2.93 | 6.66 | $448.75 | $855.58 |
| **24** | BENEWAH MEDICAL CENTER | Plummer | ID | 49.57 | 4.03 | 3.90 | $358.72 | $2,268.61 |
| **25** | FIRST NATIONS COMMUNITY HEALTHSOURCE | Albuquerque | NM | 49.29 | 0.56 | 1.44 | $190.72 | $340.27 |
| **26** | EASTERN ALEUTIAN TRIBES, INC | ANCHORAGE | AK | 45.42 | 6.02 | 3.28 | $1,221.68 | $1,503.60 |
| **27** | EASTERN ALEUTIAN TRIBES, INC | Anchorage | AK | 45.42 | 6.02 | 3.28 | $1,221.68 | $1,503.60 |
| **28** | INDIAN HEALTH BOARD OF MINNEAPOLIS, INC. | Minneapolis | MN | 45.38 | 1.39 | 3.49 | $369.90 | $1,177.31 |
| **29** | SOUTHEAST ALASKA REGIONAL HEALTH CONSORT | Sitka | AK | 43.53 | 12.84 | 0.16 | $871.08 | $922.08 |
| **30** | KARUK TRIBE OF CALIFORNIA | Happy Camp | CA | 42.67 | 6.33 | 4.48 | $388.50 | $499.33 |
| **31** | NATIVE AMERICAN COMMUNITY HEALTH CENTER, INC. | Phoenix | AZ | 38.78 | 2.92 | 3.27 | $230.99 | $818.45 |
| **32** | GLACIER COMMUNITY HEALTH CENTER, INC. | Cut Bank | MT | 35.06 | 0.87 | 2.07 | $722.15 | $726.31 |
| **33** | CANYONLANDS COMMUNITY HEALTH CARE | Page | AZ | 32.83 | 1.48 | 4.97 | $179.82 | $185.91 |
| **34** | ROBESON HEALTH CARE CORPORATION | Pembroke | NC | 28.24 | 8.52 | 2.59 | $301.58 | $438.60 |
| **35** | HORIZON HEALTH CARE, INC. | Howard | SD | 28.09 | 0.63 | 1.71 | $331.43 | $345.51 |
| **36** | BULLHOOK COMMUNITY HEALTH CENTER, INC | Havre | MT | 20.73 | 0.50 | 0.64 | $456.60 | $461.60 |

|  | | Unadjusted BPHC | | Adjusted BPHC | | Unadjusted Fed | | Adjusted Fed | |
| --- | --- | --- | --- | --- | --- | --- | --- | --- | --- |
|  | Mean | Beta | p-value | Beta | p-value | Beta | p-value | Beta | p-value |
| # Patients (Thousands) | 19.365 | -3.171 | <0.001 | -2.265 | <0.001 | -3.364 | <0.001 | -2.327 | 0 |
| % Poverty | 48.267 | -0.517 | 0.347 | 0.63 | 0.004 | -0.459 | 0.529 | 0.833 | 0.001 |
| % Uninsured | 27.808 | 3.997 | <0.001 | 1.848 | <0.001 | 4.118 | <0.001 | 1.813 | <0.001 |
| % Hispanic | 25.154 | -0.738 | 0.154 | -0.26 | 0.589 | -0.566 | 0.41 | -0.186 | 0.728 |
| % Non-Hispanic Black | 19.573 | 0.167 | 0.746 | 0.881 | 0.101 | 0.489 | 0.475 | 0.965 | 0.106 |
| % Asian | 3.101 | -0.523 | 0.648 | -1.22 | 0.375 | -0.348 | 0.819 | -1.37 | 0.371 |
| % American-Indian/Alaskan Native | 2.285 | 10.322 | <0.001 | 2.92 | 0.01 | 18.252 | <0.001 | 3.693 | 0.004 |
| % Other Racial/Ethnic Group | 6.668 | 2.906 | 0.043 | 0.962 | 0.126 | 3.34 | 0.08 | 1.184 | 0.09 |
| **Hypertension Prevalence** |  |  |  |  |  |  |  |  |  |
| Overall HTN Prevalence | 0 | 21.406 | 0.101 | 23.538 | 0.163 | 17.918 | 0.288 | 15.69 | 0.403 |
| Hispanic HTN Prevalence | 0 | -4.746 | 0.678 | 9.407 | 0.647 | -0.976 | 0.947 | 14.72 | 0.52 |
| NH Black HTN Prevalence | 0 | 4.989 | 0.709 | -30.457 | 0.362 | 12.47 | 0.47 | -11.571 | 0.756 |
| Asian HTN Prevalence | 0 | -5.498 | 0.625 | 30.086 | 0.019 | -1.647 | 0.91 | 33.828 | 0.018 |
| AI-AN HTN Prevalence | 0 | 143.442 | <0.001 | 9.97 | 0.545 | 240.421 | <0.001 | -70.262 | <0.001 |
| **Hypertension Control** |  |  |  |  |  |  |  |  |  |
| Overall HTN Control | 0 | -51.497 | 0.01 | -94.812 | <0.001 | -69.345 | 0.003 | -103.405 | <0.001 |
| Hispanic HTN Control | 0 | 27.505 | 0.08 | 41.337 | 0.005 | 19.426 | 0.287 | 38.015 | 0.022 |
| NH Black HTN Control | 0 | -4.425 | 0.692 | 37.088 | 0.001 | 20.274 | 0.119 | 48.71 | 0 |
| Asian HTN Control | 0 | 16.888 | 0.065 | 7.583 | 0.344 | 22.028 | 0.038 | 10.429 | 0.243 |
| AI-AN HTN Control | 0 | 10.283 | 0.161 | 14.255 | 0.02 | 9.771 | 0.251 | 13.909 | 0.042 |
| **Diabetes Prevalence** |  |  |  |  |  |  |  |  |  |
| Overall DM Prevalence | 0 | 22.948 | 0.134 | 6.295 | 0.718 | 25.838 | 0.187 | 12.204 | 0.53 |
| Hispanic DM Prevalence | 0 | -17.154 | 0.221 | -8.877 | 0.716 | -14.432 | 0.421 | -15.919 | 0.557 |
| NH Black DM Prevalence | 0 | 0.338 | 0.981 | 1.964 | 0.951 | 5.684 | 0.757 | -13.687 | 0.701 |
| Asian DM Prevalence | 0 | -5.171 | 0.656 | -29.135 | 0.074 | -1.898 | 0.898 | -30.173 | 0.096 |
| AI-AN DM Prevalence | 0 | 109.849 | <0.001 | -5.888 | 0.685 | 217.749 | <0.001 | 120.881 | <0.001 |
| **Diabetes Control** |  |  |  |  |  |  |  |  |  |
| Overall DM Control | 0 | 36.365 | 0.003 | 37.217 | 0.001 | 31.971 | 0.027 | 41.445 | 0.001 |
| Hispanic DM Control | 0 | -44.484 | <0.001 | -26.288 | 0.004 | -45.329 | <0.001 | -30.379 | 0.003 |
| NH Black DM Control | 0 | -29.487 | 0.002 | -36.243 | <0.001 | -29.485 | 0.008 | -37.34 | <0.001 |
| Asian DM Control | 0 | -1.61 | 0.817 | 3.191 | 0.585 | 1.467 | 0.857 | 5.23 | 0.421 |
| AI-AN DM Control | 0 | -13.071 | 0.029 | -18.007 | <0.001 | -15.433 | 0.028 | -20.538 | <0.001 |

**Sensitivity Analysis #1**

We examine the association of 2017-2019 federal grants to FQHCs with predictors from 2014-2016, including exposure variables accounting for prevalence and control of hypertension and diabetes. Prevalence terms are defined as the number of patients with hypertension or diabetes divided by total patients. Control terms are defined as the number of patients with hypertension or diabetes with adequate control divided by the number of patients with hypertension or diabetes.

**Sensitivity Analysis #2**

We stratify our original analysis by rates of uninsurance at the FQHC-level. The first analysis examines average 2017-2019 federal grants with predictors from 2014-2016, but only includes the subset of clinics (n=603) with average uninsurance rates greater than the median of all FQHCs. The second analysis examines only the subset of clinics (n=602) with uninsurance rates below the median.

| **Above Median Uninsured** | | Unadjusted BPHC | | Adjusted BPHC | | Unadjusted Fed | | Adjusted Fed | |
| --- | --- | --- | --- | --- | --- | --- | --- | --- | --- |
|  | Mean | Beta | p-value | Beta | p-value | Beta | p-value | Beta | p-value |
| # Patients (Thousands) | 17.337 | -4.293 | <0.001 | -4.197 | <0.001 | -4.556 | 0.004 | -4.422 | 0.006 |
| % Poverty | 54.036 | -1.035 | 0.342 | -0.328 | 0.763 | -1.061 | 0.467 | -0.291 | 0.842 |
| % Uninsured | 41.318 | 4.731 | 0.001 | 4.609 | 0.001 | 5.298 | 0.007 | 5.219 | 0.007 |
| % Hispanic | 31.156 | -0.741 | 0.445 | -0.973 | 0.585 | -0.668 | 0.607 | -0.836 | 0.727 |
| % Non-Hispanic Black | 22.302 | 0.122 | 0.907 | 0.091 | 0.966 | 0.462 | 0.74 | 0.69 | 0.811 |
| % Asian | 2.812 | -0.826 | 0.73 | -1.494 | 0.739 | -0.794 | 0.804 | -1.469 | 0.807 |
| % American-Indian/Alaskan Native | 2.577 | 15.651 | <0.001 | -3.117 | 0.424 | 22.665 | <0.001 | -2.257 | 0.667 |
| % Other Racial/Ethnic Group | 5.789 | 4.247 | 0.217 | 4.05 | 0.234 | 4.185 | 0.363 | 3.976 | 0.385 |
| % Overall HTN Uncontrolled | 0 | 20.633 | 0.518 | -23.125 | 0.611 | 10.818 | 0.798 | -30.334 | 0.62 |
| % Hispanic HTN Uncontrolled | 0 | -12.189 | 0.635 | 22.582 | 0.566 | -9.935 | 0.77 | 24.459 | 0.644 |
| % NH Black HTN Uncontrolled | 0 | -14.217 | 0.648 | -3.567 | 0.952 | -6.623 | 0.872 | -6.031 | 0.939 |
| % Asian HTN Uncontrolled | 0 | -10.236 | 0.621 | -4.027 | 0.91 | -11.26 | 0.682 | -1.785 | 0.97 |
| % AI-AN HTN Uncontrolled | 0 | 224.604 | <0.001 | 334.911 | <0.001 | 311.107 | <0.001 | 404.213 | <0.001 |
| % Overall DM Uncontrolled | 0 | 54.373 | 0.164 | 33.935 | 0.523 | 51.828 | 0.315 | 46.499 | 0.516 |
| % Hispanic DM Uncontrolled | 0 | -48.181 | 0.132 | -29.497 | 0.553 | -48.582 | 0.251 | -38.589 | 0.564 |
| % NH Black DM Uncontrolled | 0 | -27.302 | 0.419 | -9.974 | 0.854 | -23.013 | 0.606 | -18.755 | 0.798 |
| % Asian DM Uncontrolled | 0 | -4.941 | 0.829 | 5.705 | 0.857 | -6.05 | 0.841 | 1.171 | 0.978 |
| % AI-AN DM Uncontrolled | 0 | 160.377 | <0.001 | -104.898 | 0.012 | 238.159 | <0.001 | -94.603 | 0.09 |

| **Below Median Uninsured** | | Unadjusted BPHC | | Adjusted BPHC | | Unadjusted Fed | | Adjusted Fed | |
| --- | --- | --- | --- | --- | --- | --- | --- | --- | --- |
|  | Mean | Beta | p-value | Beta | p-value | Beta | p-value | Beta | p-value |
| # Patients (Thousands) | 21.389 | -2.7 | <0.001 | -2.553 | <0.001 | -2.836 | <0.001 | -2.656 | <0.001 |
| % Poverty | 42.508 | 0.063 | 0.829 | 0.061 | 0.833 | 0.159 | 0.692 | 0.188 | 0.583 |
| % Uninsured | 14.32 | 1.241 | 0.268 | 1.861 | 0.101 | 0.707 | 0.646 | 1.585 | 0.232 |
| % Hispanic | 19.163 | -0.316 | 0.308 | -0.925 | 0.176 | -0.019 | 0.965 | -0.469 | 0.558 |
| % Non-Hispanic Black | 16.848 | 0.413 | 0.137 | 1.293 | 0.027 | 0.715 | 0.061 | 1.288 | 0.06 |
| % Asian | 3.389 | -0.495 | 0.394 | -0.337 | 0.793 | -0.22 | 0.783 | -2.044 | 0.175 |
| % American-Indian/Alaskan Native | 1.994 | 2.925 | <0.001 | 3.077 | 0.024 | 12.18 | <0.001 | -6.096 | <0.001 |
| % Other Racial/Ethnic Group | 7.545 | 1.414 | 0.038 | 1.757 | 0.012 | 2.025 | 0.031 | 2.115 | 0.01 |
| % Overall HTN Uncontrolled | 0 | 19.957 | 0.01 | 19.181 | 0.042 | 9.997 | 0.323 | 19.446 | 0.078 |
| % Hispanic HTN Uncontrolled | 0 | -18.267 | 0.006 | 2.372 | 0.829 | -15.454 | 0.074 | 1.304 | 0.919 |
| % NH Black HTN Uncontrolled | 0 | 0.484 | 0.95 | -21.001 | 0.098 | 9.687 | 0.337 | -18.788 | 0.205 |
| % Asian HTN Uncontrolled | 0 | -3.479 | 0.602 | 4.883 | 0.608 | 1.166 | 0.894 | 7.891 | 0.48 |
| % AI-AN HTN Uncontrolled | 0 | 33.379 | <0.001 | 19.253 | 0.079 | 125.494 | <0.001 | 53.575 | <0.001 |
| % Overall DM Uncontrolled | 0 | 28.514 | 0.003 | 20.62 | 0.061 | 24.843 | 0.024 | 13.756 | 0.285 |
| % Hispanic DM Uncontrolled | 0 | -25.325 | 0.002 | 12.92 | 0.353 | -22.112 | 0.015 | 7.218 | 0.658 |
| % NH Black DM Uncontrolled | 0 | -1.391 | 0.869 | -12.128 | 0.324 | 8.432 | 0.382 | -3.734 | 0.795 |
| % Asian DM Uncontrolled | 0 | -3.818 | 0.572 | -5.788 | 0.612 | 7.15 | 0.354 | 17.86 | 0.182 |
| % AI-AN DM Uncontrolled | 0 | 15.911 | 0.025 | -23.865 | 0.025 | 153.572 | <0.001 | 160.492 | <0.001 |
